# Complete chloroplast genome sequence of *Kosteletzkya pentacarpos*

**DOI:** 10.1080/23802359.2022.2093665

**Published:** 2022-07-06

**Authors:** Zhiquan Wang, Hong Yang, Fengjiao Zhang, Yunlong Yin, Chunsun Gu

**Affiliations:** a Institute of Botany, Jiangsu Province and Chinese Academy of Sciences, Nanjing, China; b Jiangsu Provincial Platform for Conservation and Utilization of Agricultural Germplasm, Jiangsu Key Laboratory for the Research and Utilization of Plant Resources, Nanjing, China; c College of Forest Sciences, Nanjing Forestry University, Nanjing, China

**Keywords:** *Kosteletzkya pentacarpos*, complete chloroplast genome, phylogenetic analysis

## Abstract

*Kosteletzkya pentacarpos* is a promising plant being developed as a salt-tolerant biofuel crop that also the ability to fix heavy metals. Here, high-throughput sequencing technology was used to sequence and assemble the chloroplast genome of *K. pentacarpos*. The full length of the chloroplast genome is 161,777 bp, comprising a large single-copy region of 90,019 bp, a small single-copy region of 18,978 bp, and a pair of inverted repeats of 26,390 bp. A total of 113 genes were annotated, including 79 protein-coding, 30 transfer RNA, and 4 ribosomal RNA genes. Phylogenetic analysis based on whole chloroplast genome sequences showed that *K. pentacarpos* has a close relationship with *Abelmoschus* in Malvaceae. This study increases the available genomic information on *K. pentacarpos*, and provides a basis for the rational exploitation and utilization of germplasm resources.

*Kosteletzkya pentacarpos* (Linnaeus) Ledebour 1842 is a perennial dicot in the family Malvaceae that is being developed as a salt-tolerant biofuel crop for salt-affected coastal land (Halchak et al. 2011; Moser et al. 2013). Its cultivation could prolong the economic viability of the land (Gallagher 1985; Voutsina 2012). This plant also has the ability to fix heavy metals through the mucilage, as has recently been demonstrated for copper, cadmium, and zinc (Lutts et al. 2016; Zhou et al. 2019; 2020). However, there are limited reports that clarify the phylogeny of this promising plant. As an effective DNA molecular marker, the chloroplast genome has been widely used in genetic and evolutionary relationship studies in plants (Freitas et al. 2018). Here, using Illumina sequencing, we determined and assembled the complete chloroplast genome sequence of *K. pentacarpos*, resulting in more sequence data, which can be used to understand the genomes and phylogenetic relationships among Malvaceae family members.

The sample of *K. pentacarpos* was collected from Nanjing Botanical Garden, Mem. Sun Yat-sen (118°49′55″E, 32°3′32″N), Nanjing, China. Total genomic DNA was extracted using a plant DNA isolation reagent (Code: D9194, TaKaRa, Dalian, China) from the fresh mature leaves of *K. pentacarpos* leaves. The specimen and DNA were deposited in Nanjing Botanical Garden, Mem. Sun Yat-sen (Zhiquan Wang, wangzhiquan@cnbg.net) under voucher number NBG-KP-0001. A paired-end library with an insert-size of 350 bp was constructed and sequenced on an Illumina NovaSeq 6000 system (Illumina, San Diego, CA, USA). In total, approximately 8 Gb of clean data (25,236,586 reads) were obtained. The chloroplast genome was assembled using the GETORGANELLE pipeline (Jin et al. 2020) and annotated using Geneious Prime v.2021.1.1 (http://www.geneious.com), with *Hibiscus cannabinus* (NC_045873) as a reference. It was validated using CPGAVAS2 (Shi et al. 2019).

The sequence of the *K. pentacarpos* genome was deposited in GenBank (accession. OK336488). The *K. pentacarpos* chloroplast genome was determined to be 161,777 bp in length, including a large single-copy region of 90,019 bp and a small single-copy region of 18,978 bp separated by two inverted repeats of 26,390 bp. The overall GC content of the chloroplast genome is 36.9%. The genome contains 113 genes, including 79 protein-coding, 30 transfer RNA, and 4 ribosomal RNA genes. Seven protein-coding, seven transfer RNA, and four ribosomal RNA genes are duplicated in the inverted repeat region. There are 17 chloroplast genes harboring introns, with 15 containing single introns and two (*ycf3* and *clpP*) containing two introns.

The complete chloroplast genome sequences of *K. pentacarpos* and 10 other species of *Hibisceae* were used for a phylogenetic analysis, with *Gossypium herbaceum* (JF317353) as an outgroup. The chloroplast genome sequences were aligned using the MAFFT plugin (Katoh and Standley 2013) as implemented in Geneious Prime v.2021.1.1 (Biomatters Ltd., Auckland, New Zealand). The best-fitting model of nucleotide substitutions was determined using the Akaike information criterion in jModelTest v2.1.4 (Posada 2008), and the GTR + G + I substitution model was selected. Maximum-likelihood analysis was conducted using RAxML-HPC v.8.2.8 (Stamatakis 2014) with 1,000 bootstrap replicates on the CIPRES Science Gateway website (https://www. phylo.org/). The phylogeny revealed that *K. pentacarpos* is a sister to the species of *Abelmoschus* L. (Figure 1). The chloroplast genome sequence of *K. pentacarpos* determined in this study will be useful for further analyses of molecular markers and molecular breeding.

**Figure 1.**
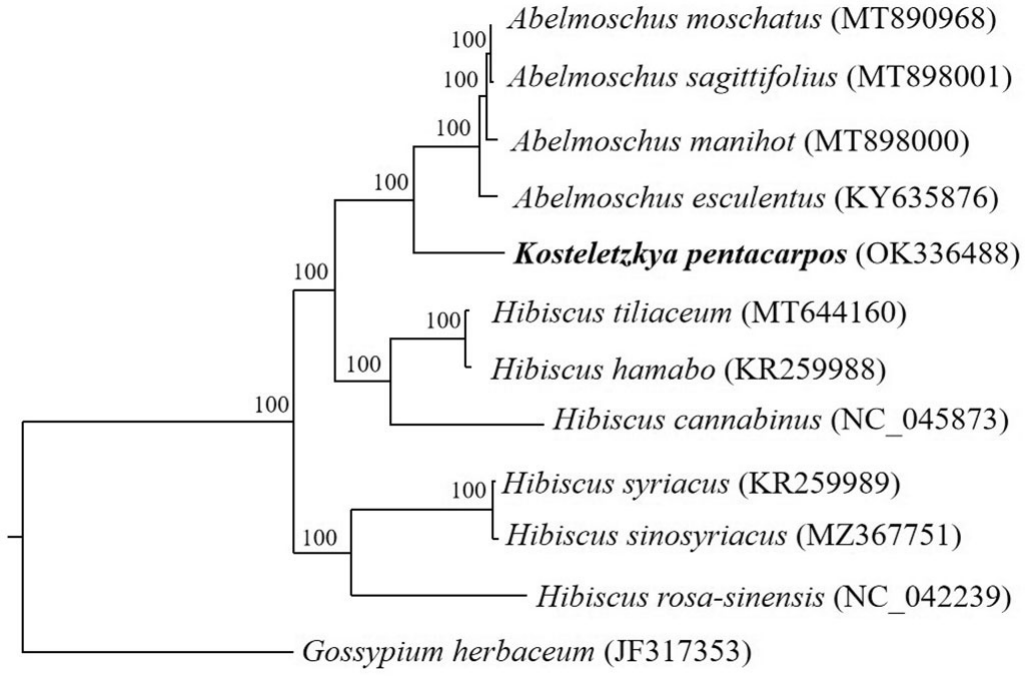
Phylogenetic tree, based on the chloroplast genome sequences of 12 species, showing the close relationship between *K. pentacarpos* and *Abelmoschus* L. Numbers next to the nodes indicate the bootstrap value from 1,000 replicates. The GenBank accession number for each species is shown in the parentheses after the name.

## Data Availability

The genome sequence data that support the findings of this study are openly available in GenBank of NCBI at [https://www.ncbi.nlm.nih.gov] under accession no. OK336488. The associated BioProject, SRA, and Bio-Sample numbers are PRJNA767249, SRR16114382, and SAMN21895023, respectively.
